# Emerging Roles of Hydrogen Sulfide in Inflammatory and Neoplastic Colonic Diseases

**DOI:** 10.3389/fphys.2016.00156

**Published:** 2016-05-03

**Authors:** Fang-Fang Guo, Ta-Chung Yu, Jie Hong, Jing-Yuan Fang

**Affiliations:** State Key Laboratory of Oncogene and Related Genes, Key Laboratory of Gastroenterology and Hepatology, Division of Gastroenterology and Hepatology, Ministry of Health, School of Medicine, Shanghai Cancer Institute, Shanghai Institution of Digestive Disease, Ren Ji Hospital, Shanghai Jiao Tong UniversityShanghai, China

**Keywords:** hydrogen sulfide, sulfate-reducing bacteria, pathophysiological roles, colonic diseases, chemical target

## Abstract

Hydrogen sulfide (H_2_S) is a toxic gas that has been recognized as an important mediator of many physiological processes, such as neurodegeneration, regulation of inflammation, blood pressure, and metabolism. In the human colon, H_2_S is produced by both endogenous enzymes and sulfate-reducing bacteria (SRB). H_2_S is involved in the physiological and pathophysiological conditions of the colon, such as inflammatory bowel disease (IBD) and colorectal cancer (CRC), which makes the pharmacological modulation of H_2_S production and metabolism a potential chemical target for the treatment of colonic diseases. However, the exact mechanisms and pathways by which H_2_S-mediates normal physiological function and disease in the colon are not fully understood. Besides, the production and release of H_2_S are modulated by both endogenous and exogenous factors. This review will discuss the production and storage of H_2_S, its biological roles and the emerging importance in physiology and pathology of IBD and CRC.

## Introduction

Hydrogen sulfide (H_2_S) is a pungent gas that smells like rotten eggs, and has been identified as the third gaseous transmitter, following nitric oxide (NO) and carbon monoxide (CO; Gallego et al., 2008). Since the discovery of its synthesis in mammalian and human tissues, it has attracted much interest as an endogenous mediator in recent years (Whiteman et al., 2011). Over the last decade, H_2_S has been recognized to have various biological effects in human health and diseases, such as in the nervous system, the cardiovascular system, and the immune system (Kimura, 2011; Wang et al., 2012). Recently, studies involving the physiological and pathophysiological effects of H_2_S in the gastrointestinal tract (GI tract) have attracted much attention. Multiple studies also imply the important role of H_2_S in colonic diseases, including inflammatory bowel disease (IBD; Wallace et al., 2009; Hirata et al., 2011) and colorectal cancer (CRC) (Cai et al., 2010; Cao et al., 2010; Kimura, 2011). In the present review, we will discuss the endogenous and exogenous production of H_2_S, and its biological and pathological roles in IBD and CRC.

## Endogenous production and biological roles of H_2_S

The concentration of H_2_S ranges from 0.2 to 1 mmol/L in the colon of mice and may reach 3.4 mmol/L in human stools (Rose et al., 2005). Under normal conditions, approximately 70% of H_2_S is produced from cysteine and the other 30% from homocysteine (Chiku et al., 2009). There are three principal enzymes involved in the endogenous production of H_2_S: cystathionine β-synthase (CBS), cystathionine γ-lyase (CSE) and 3-mercaptopyruvate sulfurtransferase (3-MST). They are expressed in many organs, including the liver, kidney, ileum, and brain (Kimura, 2011). CBS and CSE have been investigated widely, and both use vitamin B6 as a cofactor to catalyze the production of H_2_S (Chiku et al., 2009). The catalytic effect of CBS changes with the extent of allosteric activation of S-adenosylmethionine (Singh et al., 2009), and the activity of CSE is enhanced by sodium nitroprusside (SNP; Chiku et al., 2009). A study also shows that CSE is regulated by calcium calmodulin, although the requirement for Ca^2+^ concentrations is quite high (1 mM; Yang et al., 2008). The role of 3-MST along with cysteine aminotransferase (CAT), which can efficiently produces H_2_S from cysteine and a-ketoglutarate (Kimura, 2011), in regulating endogenous H_2_S levels has recently been examined in specific types of cells and tissues (Shibuya et al., 2009b; Wang, 2012).

H_2_S may function as a signal molecule immediately after released from the enzyme; it can also be stored as bound sulfane sulfur, which may in turn release H_2_S (Whiteman et al., 2011). At physiological pH, nearly two-thirds of H_2_S exists as the hydrosulfide anion (HS^−^), which is a powerful nucleophile (Bouillaud and Blachier, 2011).

**Figure 1:**
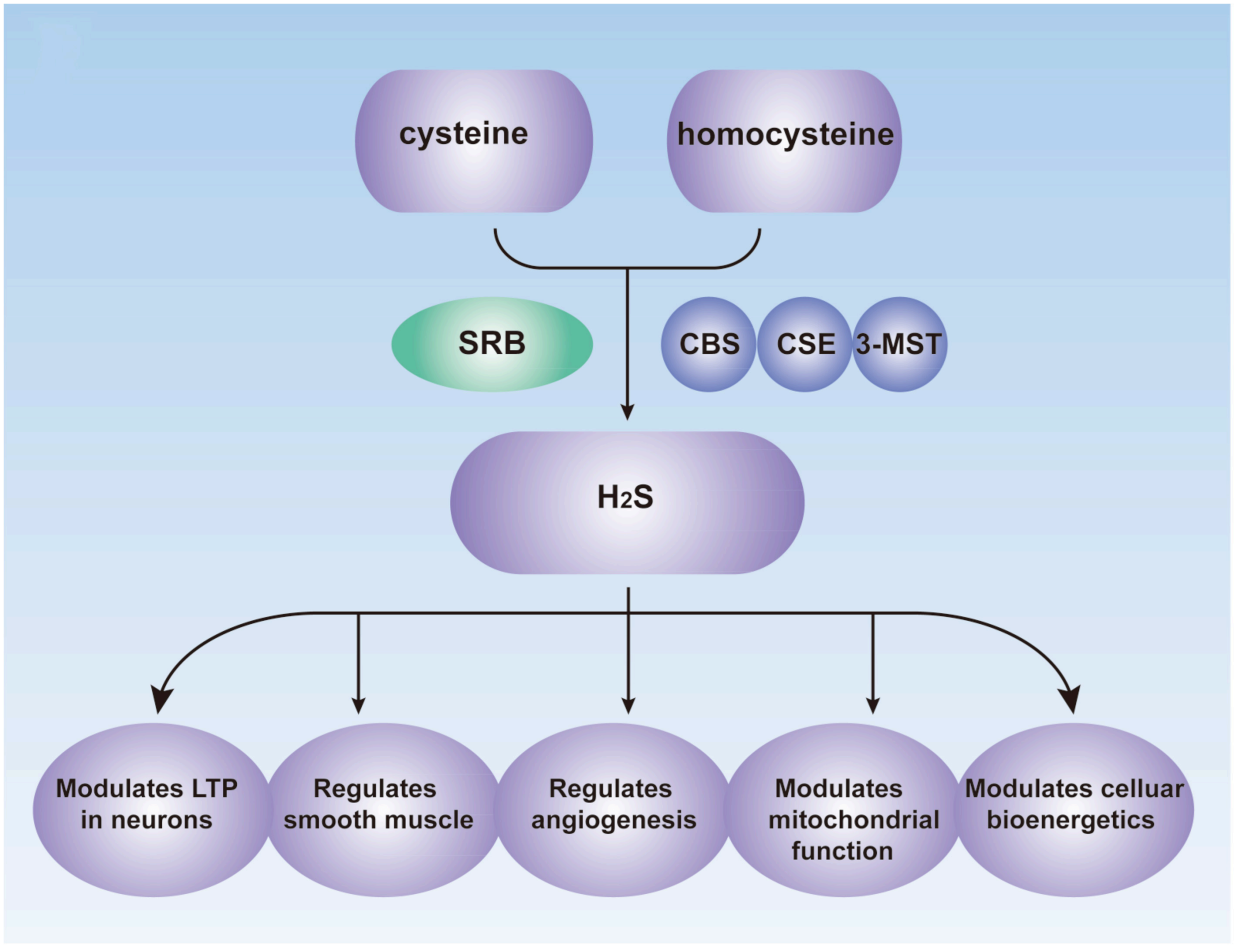
**Biological Roles of H_2_S in the human**. CBS, cystathionine β-synthase; CSE, cystathionine γ-lyase; 3-MST, 3-mercaptopyruvate sulfurtransferase; SRB, sulfate-reducing bacteria; LTP, long-term potentiation.

Endogenous H_2_S performs vital roles in many physiological processes, including vasorelaxation, angiogenesis, cellular energy production, neuromodulation, cytoprotection, and pathological processes (Figure 1; Kimura et al., 2010; Coletta et al., 2012), and it is now considered as a signaling modulator or a messenger molecule (Farrugia and Szurszewski, 2014). H_2_S was initially considered as a neuromodulator that aids the induction of hippocampal long-term potentiation (LTP) by enhancing NMDA-induced currents in neurons (Abe and Kimura, 1996; Nagai et al., 2004). H_2_S may also mediate the reciprocal interactions between glial calcium waves and neuronal activity, which has not been fully investigated (Kimura, 2011). Prior studies also showed that transient receptor potential (TRP) channels might be involved in the effects of H_2_S (Patacchini et al., 2005; Gratzke et al., 2009).

H_2_S also functions as signal molecule in smooth muscle relaxation. Although NO performs most of the vessel-relaxing work in large vessels, H_2_S may be responsible for similar actions in smaller blood vessels (Wang, 2009). The mechanisms of H_2_S-mediated vasodilation may involve the activation of K_ATP_ channels or other channels, the inhibition of phosphodiesterases and synergy with NO (Wang, 2012).

The important pro-angiogenic role of H_2_S has also been recognized (Coletta et al., 2012). Angiogenesis is a complex biological process involved in endothelial cell proliferation, migration, and formation of capillary structures (Roudsari and West, 2015). Pupo et al. showed that endogenous H_2_S is involved in the angiogenic effects of vascular endothelial growth factor (VEGF), a key growth factor and tumor-derived angiogenic hormone (Pupo et al., 2011). Other studies showed that H_2_S exerts its effects via multiple mechanisms including activation of VEGFR2, stimulation of potassium channels and increase of cellular glutathione (GSH) levels (Cai et al., 2010; Cao et al., 2010; Kimura et al., 2010; Tao et al., 2013).

In addition to serving as a signal molecule, H_2_S also participates in concentration-dependent modulation of mitochondrial function and cellular bioenergetics. In various cell types (including intestinal epithelial cells and hepatocytes), low concentrations of H_2_S act as mitochondrial electron donors, which results in the stimulation of bioenergy (Szabo et al., 2014). They increase the levels of glutathione and redistribute it to the mitochondria. In addition, they can promote the catalytic activity of the glycolytic enzyme GAPDH (Mustafa et al., 2009). Endogenous H_2_S may also serve as a bioenergetic stimulator (Modis et al., 2013a,c). H_2_S produced by 3-MST along with CAT can scavenge reactive oxygen species in mitochondria and protect cells from oxidative stress (Kimura et al., 2010). Modis et al. demonstrated that H_2_S donors could stimulate mitochondrial electron transport and ATP generation in various cell lines *in vitro* (Modis et al., 2013b).

However, when the concentrations of this molecule are relatively high, the stimulatory effect of H_2_S is superseded by an inhibitory effect (Szabo et al., 2014), and high concentration of H_2_S may become a broad-spectrum poison to the nervous system, respiratory system and cardiovascular system (Wang, 2012). The concentration of H_2_S produced naturally in the human body is much lower than the toxic levels, which may be necessary for cell survival (Kimura, 2011).

The complexities of H_2_S biology may be related to its pharmacology. It is a diffusible gas and has a bell-shaped or biphasic dose-response curve, whereby lower concentrations of H_2_S show quite different (often, opposing) effects compared with higher concentrations (Szabo et al., 2013). Lower levels of H_2_S exert multiple physiological, cytoprotective, antioxidant and, anti-inflammatory functions. At higher local levels, however, H_2_S can become prooxidant, cytostatic, and cytotoxic (Baskar and Bian, 2011). However, these studies are limited by the lack of enzyme-specific inhibitors to target H_2_S biosynthesis, which may be related to the above-mentioned controversial observations (Whiteman et al., 2011).

## Biological roles of H_2_S in the GI tract

Emerging evidence indicate that endogenous H_2_S can be produced and released by colonic tissue (Linden et al., 2008; Cao et al., 2010). In the GI tract H_2_S is mainly produced by CBS and CSE. CSE seems to be the main H_2_S-generating enzyme in the stomach, while CBS is the major enzyme in the colon (Wallace et al., 2009). The functions of H_2_S in the GI tract have also received much attention in recent years. It can relax ileal smooth muscle, increase colonic secretion (Gallego et al., 2008; Matsunami et al., 2009), and protect the intestines from ischemia–reperfusion injury in rats (Liu et al., 2009). However, high levels of H_2_S may also cause diseases, such as IBD and CRC.

## Roles of exogenous H_2_S donors

There are multiple reports related to exogenous H_2_S donors in tumor cells that either promote or inhibit cell proliferation at different concentrations (Baskar and Bian, 2011; Wu et al., 2012). Previous studies have attempted to use various molecules to produce H_2_S (Kashfi and Olson, 2013), such as NSAIDs (Chattopadhyay et al., 2013; Kashfi, 2014), GYY4137 [morpholin-4-ium 4 methoxyphenyl (morpholino)] (Ning et al., 2014), S-propargyl-cysteine (Ma et al., 2011), Sodium hydrosulfide (NaHS) (Cai et al., 2010), Na_2_S (Hirata et al., 2011). The pros and cons of these molecules are summarized in a review article by Hellmich et al. (2015). Among them NaHS is most widely used to study the physiological functions of H_2_S. NaHS is a fast-release H_2_S donor, which immediately dissociates and forms the hydrosulfide anion (HS^−^) in the liquid culture, and reacts with H^+^ to form H_2_S.

Many studies have reported duplex effects of H_2_S on cell proliferation/cell death in various transformed and non-transformed cell lines *in vitro* (Leschelle et al., 2005; Cai et al., 2010; Murata et al., 2014). Baskar et al. have summarized most of these reports in a review article (Baskar and Bian, 2011). Note that the effects of H_2_S donors are bi-phasic, just like endogenously produced H_2_S. Cai et al. reported a concentration-dependent stimulation of cell growth by NaHS at doses between 10 and 50 μM, a plateauing of the effect at 200 μM, and an inhibition of proliferation at 1000 μM in HCT116 and SW480 cells (Cai et al., 2010). Hellmich et al. also demonstrated that the nature of the cellular response (stimulation or inhibition of growth) is determined by the rate of H_2_S production (fast- vs. slow-release H_2_S donors) as well as by the concentration of donor relative to the basal level of endogenous enzyme-dependent H_2_S production (Hellmich et al., 2015). It should be considered that H_2_S donors with different release rates might induce quantitative, as well as qualitative, variance in cellular responses (Whiteman et al., 2010; Baskar and Bian, 2011).

Thus, the bell-shaped properties of H_2_S provide a useful framework to reconcile some of the controversies regarding H_2_S functions. However, the complexities of the temporal relationship between H_2_S donation and its effects remain to be further explored.

## Bacteria associated with hydrogen sulfide metabolism

H_2_S was one of the earliest products of bacterial decomposition to be recognized (Shatalin et al., 2011). Sulfur reduction and oxidation are handled by two different groups of bacteria. The former comprise the sulfate-reducing bacteria (SRB) and sulfur-reducing bacteria, while the latter includes sulfur-oxidizing bacteria and sulfide-oxidizing bacteria (Wang, 2012). They both contribute to a balanced H_2_S level in a given environment. Among them, SRB belong to the most ancient forms of bacteria and utilize a wide range of substrates, including hydrogen, short-chain fatty acids, alcohols and amino acids to reduce sulfur and sulfur-containing compounds to H_2_S (Scanlan et al., 2009).

SRB are Gram-negative, non-spore-forming, obligate anaerobes. SRB are considered to be strictly anaerobic microorganisms, but they are also found in anoxic habitats depleted of sulfate, such as the GI tract (Rey et al., 2013). Although Hansen et al. demonstrated the lack of SRB in human gut microbiota (Hansen et al., 2011), other studies using different analytic approaches have identified SRB in the fecal microbiota of healthy adults and the distal gut mucosa (Stewart et al., 2006; Rey et al., 2013). The number of healthy individuals harboring SRB ranged from 24 to 100% (Loubinoux et al., 2002). The most frequently detected SRB from animal and human feces that are relevant to bowel colonization are *flagellate Vibrio bacteria* and *Desulfovibrio* (Scanlan et al., 2009).

For bacterial-derived H_2_S, little is known about the metabolic pathways involving host cellular processes. Huycke et al. showed that H_2_S produced by SRB in the GI tract is potentially genotoxic to the gut epithelium (Huycke and Gaskins, 2004). However, Konstantin et al. demonstrated that H_2_S produced by SRB acts as a cytoprotectant molecule against oxidative stress and antimicrobials by suppressing the DNA-damaging Fenton reaction and stimulating the major antioxidant enzymes catalase and superoxide dismutase (SOD) (Shatalin et al., 2011). Moreover, Devkota et al. found that SRB are positively associated with inflammation (Devkota et al., 2012): both pro- and anti-inflammatory signaling is attributed to H_2_S (Pitcher et al., 2000; Wallace et al., 2009). In conclusion, bacterial-derived H_2_S may have important roles in the GI tract, but the conclusions remain to be further explored.

## Roles of H_2_S in the pathophysiology of IBD and CRC

### IBD

The incidence of IBD and other immune-related human disorders have increased considerably over the past 50 years, matching the changes in human diet and lifestyle. The pathological roles of colonic luminal H_2_S and/or SRB in IBD have attracted much attention recently (Roediger et al., 1997; Wallace et al., 2009; Hirata et al., 2011). However, the viewpoint that H_2_S contributes to the pathogenesis of IBD remains controversial (Table 1). It has been reported previously that high levels of H_2_S produced by bacteria could contribute to ulcerative colitis (UC) by damaging oxidation of n-butyrate, leading to impaired barrier function (Levitt et al., 2002). However, several studies have challenged the idea for the lack of compelling evidence that H_2_S causes damage to colonic epithelial cells, and have demonstrated that H_2_S can act as a metabolic fuel for colonocytes (Goubern et al., 2007; Picton et al., 2007). Wallace et al. reported significant accumulation of H_2_S after induction of colitis in rats and inhibition of H_2_S synthesis exacerbates colitis, suggesting that H_2_S contributes to the resolution of experimental colitis (Wallace et al., 2009). Recently, Hirata et al. also confirmed that endogenous H_2_S acted as an anti-inflammatory molecule by preventing neutrophil accumulation and via its anti-oxidant ability, suggesting cytoprotective effects of H_2_S (Hirata et al., 2011).

**Table 1:** **Roles of H_2_S in the pathophysiology of IBD and CRC**.

**Diseases**	**Effects of H_2_S**	**Possible pathogenesis/Epidemiologic study**	**References**
IBD	Pro-inflammatory effects	Impaired oxidation of n-butyrate	Levitt et al., 2002
		Patients with UC had excessive SRB colonization or H2S in feces	Pitcher et al., 2000; Rowan et al., 2009
	Anti-infammatory effects	Suppression of the activation of NF-kB	Oh et al., 2006
		Promotion of ucler healing in rats	Wallace et al., 2007b
		Downregulation of TNF-α,IFN-γ and iNOS epression	Li et al., 2007; Wallace et al., 2007a
		Contribution to the resolution of experimental colitis	Wallace et al., 2009
		Acting as an antioxidant	Hirata et al., 2011
		Preventation of neutrophil accumulation and viaits anti-oxidant ability	Hirata et al., 2011
	No effects	No difference in SRB between patients with IBD and controls	Fite et al., 2004; Picton et al., 2007
CRC	Carcinogenic factor	Decrease of suifide-detoxifying enzymes	Ramasamy et al., 2006
		Genomic DNA damage	Attene-Ramos et al., 2007
		Stimulation of the growth and migration	Cai et al., 2010; Szabo et al., 2013; Modis et al., 2014
		Inhibition of cell apoptosis	Sen et al., 2012
		Stimulation of tumor angiogenesis and peritumoral vasodilation	Szabo et al., 2013
	Cancer suppressive factor	Reduction of cell viability	Cao et al., 2010
		Inhibition of proliferation and promotion of protective autophagy	Wu et al., 2012

Although the functions of endogenous and exogenous H_2_S in IBD remains controversial, many prior studies have shown multiple effects of H_2_S. It can downregulate the expression of several pro-inflammatory cytokines and enzymes, such as TNF-α, IFN-γ and iNOS (Li et al., 2007; Wallace et al., 2007a); suppress the activation of NF-κB (Oh et al., 2006); act as an antioxidant (Hirata et al., 2011); and promote ulcer healing in rats (Wallace et al., 2007b). The colonic mucosa is endowed with an efficient H_2_S-detoxifying mechanism, oxidizing more than 300 μmol of H_2_S daily in the rat colon (Suarez et al., 1998). When the barrier breaks down, such as in severe colitis, a large amount of H_2_S may access the muscle layers and inhibit motility.

In addition, the contribution of bacteria-derived H_2_S in colitis remains unclear. An epidemiologic study revealed that patients suffering from UC had either excessive SRB colonization or excessive H_2_S in their feces (Pitcher et al., 2000; Rowan et al., 2009). Kleessen et al. reported variable counts of SRB from colonic mucosal specimens in patients with UC, Crohn's disease (CD) and healthy controls (Kleessen et al., 2002). However, Picton et al. found no evidence of defective enzymic detoxication of sulfide in patients with UC or CD (Picton et al., 2007). Fite et al. also confirmed that there is no disease-related difference in SRB carriage between patients with UC and controls by rectal biopsies (Fite et al., 2004).

In summary, endogenously and exogenously produced H_2_S in the GI tract might contribute to colitis and IBD, but there is no complete mechanistic model that explains the relationship.

### CRC

CRC is the second leading cause of death from cancer and the fourth most common cancer in men and women worldwide. H_2_S is also implicated in CRC (Huycke and Gaskins, 2004; Cai et al., 2010; Cao et al., 2010; Hellmich and Szabo, 2015). Although the basal expression of H_2_S-synthesizing enzymes in human colon tissue is relatively low (Whiteman et al., 2011), Szabo et al. observed the selective upregulation of CBS in the colon cancer tissue compared to normal mucosa tissue (Szabo et al., 2013). The expression of CBS is also upregulated in certain colon adenocarcinoma-derived cell lines (HCT-116, HT-29, and LoVo) compared with the colonic epithelial cell line. (Szabo and Hellmich, 2013) Genomic DNA damage is observed in colon cells after H_2_S exposure (Attene-Ramos et al., 2007). In addition, sulfide-detoxifying enzymes in the human colon are decreased in cancer tissues (Ramasamy et al., 2006).

Recently, several studies suggested that H_2_S regulated cell growth or death in a multitude of settings (Cai et al., 2010; Cao et al., 2010; Medani et al., 2011; Szabo et al., 2013). Cai et al. demonstrated that H_2_S promoted colon cancer cell proliferation, as mentioned previously (Cai et al., 2010). However, Cao et al. demonstrated that H_2_S is endogenously produced in colonic tissues and that exogenously applied H_2_S at physiologically concentrations reduced cell viability (Cao et al., 2010). Another study showed that H_2_S could inhibit proliferation and promote protective autophagy in colon epithelial cells by the activation of the AMPK/ mTOR cascade (Wu et al., 2012). Previous studies have shown cell type-specific activation of MAPK by H_2_S, which determines the fates of cells (Cho et al., 2006; Shibuya et al., 2009a; Cao et al., 2010). These controversies may relate to the bell-shaped dose-response curve of H_2_S.

For tumor-produced CBS-derived H_2_S in CRC, Szabo et al. defined this gas as a combined autocrine and paracrine-signaling molecule (Szabo and Hellmich, 2013). As an autocrine factor, H_2_S stimulates the proliferation and migration of CRC cells (Cai et al., 2010; Szabo and Hellmich, 2013; Szabo et al., 2013; Modis et al., 2014). However, at higher concentrations or longer exposures to S-adenosyl-L-methionine (SAM), the inhibitory effects become more prominent because of cytotoxicity. Recently, Sen et al. found that the sulfhydration of nuclear factor kappa B (NF-kB) by H_2_S could inhibit cell apoptosis (Sen et al., 2012). The mechanisms of the proliferative and pro-migratory effects might be the stimulation of the Akt/PI3K signaling pathway, decrease of p21 gene expression and interaction with NO (Cai et al., 2010).

As a paracrine factor, H_2_S might diffuse out from the tumor cell to stimulate tumor angiogenesis and peritumoral vasodilation. Szabo et al. reported that treatment of nude mice with a CBS inhibitor could attenuate the growth of patient-derived colon cancer xenografts and reduce peritumoral blood flow (Szabo et al., 2013). This study also confirmed the stimulatory role of H_2_S on the activity of GAPDH, indicating that H_2_S can affect both oxidative and glycolytic metabolism in tumor cells. Another independent study also confirmed the autocrine and paracrine functions of colon cancer-derived H_2_S (Yamagishi et al., 2012). Yamagishi et al. detected significant amounts of H_2_S inside colon cancer tissue.

Subsequent studies in nude mice bearing xenografts of either HCT116 or patient-derived tumor tissue (PDTX) extended the findings into *in vivo* models. Inhibition of CBS significantly reduced the growth rate of the tumor xenografts, which might be related to intratumoral mechanisms or paracrine mechanisms in the tumor microenvironment (Hellmich and Szabo, 2015). In addition, CSE can stimulate colon cancer cell proliferation, migration *in vitro* and tumor xenografts growth *in vivo*, however, the roles of CSE/H_2_S in colon cancer remain uncertain. Another study demonstrated that the canonical Wnt pathway can upregulate CSE expression (Fan et al., 2014).

Thus, H_2_S may exhibit both protective and pathological effects in the GI tract given its biphasic pharmacological characters. However, the prior studies demonstrated controversial effects of H_2_S and the mechanisms remain unknown.

## The therapeutic potential of H_2_S in colonic diseases

Although limited in terms of quantity and mechanistic models, there is reasonable evidence suggesting that H_2_S is important for the occurrence and development of colonic diseases. The intriguing discovery that H_2_S governs specific protective responses against oxidative stress and antibiotics also suggested the potential therapeutic implications (Shatalin et al., 2011). We can hypothesize that H_2_S inhibition might be potentially applicable to inhibition of tumor blood supply and/or the hyperproliferative response in CRC. Given the particular pharmacological character of H_2_S, both stimulation and inhibition of H_2_S might have potential therapeutic applications (Szabo and Papapetropoulos, 2011).

In addition, it is noted that exposure to a relatively low level of H_2_S over a relatively long time period selectively inhibits cancer cell proliferation. Therefore, slow-releasing H_2_S donors and H_2_S-releasing hybrid drugs could be designed and developed as novel anticancer drugs (Wu et al., 2015). However, these possibilities are merely hypothetical at present, and the lack of wholly enzyme- and tissue-specific inhibitors of H_2_S has meant that controversial or contradictory conclusions have been made in previous studies.

In conclusion, H_2_S might play vital roles in the development of colonic diseases, and further investigations are needed to determine the proper dose range and time frame of H_2_S in IBD and CRC, thereby achieving optimal anti-inflammation and anti-cancer effects.

## Author contributions

FG collected the references and wrote the majority of the manuscript; TY helped with the preparation and the revision of the manuscript; JH contributed to the correction of the grammar and terminology; JF contributed to the final version of the manuscript.

### Conflict of interest statement

The authors declare that the research was conducted in the absence of any commercial or financial relationships that could be construed as a potential conflict of interest.

## References

[B1] Abe K. Kimura H. (1996). The possible role of hydrogen sulfide as an endogenous neuromodulator. J. Neurosci. 16, 1066–1071. 8558235 10.1523/JNEUROSCI.16-03-01066.1996 PMC6578817

[B2] Attene-Ramos M. S. Wagner E. D. Gaskins H. R. Plewa M. J. (2007). Hydrogen sulfide induces direct radical-associated DNA damage. Mol. Cancer Res. 5, 455–459. 10.1158/1541-7786.MCR-06-0439 17475672

[B3] Baskar R. Bian J. (2011). Hydrogen sulfide gas has cell growth regulatory role. Eur. J. Pharmacol. 656, 5–9. 10.1016/j.ejphar.2011.01.052 21300051

[B4] Bouillaud F. Blachier F. (2011). Mitochondria and sulfide: a very old story of poisoning, feeding, and signaling? Antioxid. Redox Signal. 15, 379–391. 10.1089/ars.2010.3678 21028947

[B5] Cai W. J. Wang M. J. Ju L. H. Wang C. Zhu Y. C. (2010). Hydrogen sulfide induces human colon cancer cell proliferation: role of Akt, ERK and p21. Cell Biol. Int. 34, 565–572. 10.1042/CBI20090368 20184555

[B6] Cao Q. Zhang L. Yang G. Xu C. Wang R. (2010). Butyrate-stimulated H2S production in colon cancer cells. Antioxid. Redox Signal. 12, 1101–1109. 10.1089/ars.2009.2915 19803745

[B7] Chattopadhyay M. Nath N. Kodela R. Sobocki T. Metkar S. Gan Z. Y.. (2013). Hydrogen sulfide-releasing aspirin inhibits the growth of leukemic Jurkat cells and modulates beta-catenin expression. Leuk. Res. 37, 1302–1308. 10.1016/j.leukres.2013.07.004 23896061 PMC3769470

[B8] Chiku T. Padovani D. Zhu W. Singh S. Vitvitsky V. Banerjee R. (2009). H2S biogenesis by human cystathionine gamma-lyase leads to the novel sulfur metabolites lanthionine and homolanthionine and is responsive to the grade of hyperhomocysteinemia. J. Biol. Chem. 284, 11601–11612. 10.1074/jbc.M808026200 19261609 PMC2670165

[B9] Cho S. D. Ahn N. S. Jung J. W. Yang S. R. Park J. S. Lee Y. S.. (2006). Critical role of the c-JunNH2-terminal kinase and p38 mitogen-activated protein kinase pathways on sodium butyrate-induced apoptosis in DU145 human prostate cancer cells. Eur. J. Cancer Prev. 15, 57–63. 10.1097/01.cej.0000195704.05246.fc 16374231

[B10] Coletta C. Papapetropoulos A. Erdelyi K. Olah G. Modis K. Panopoulos P.. (2012). Hydrogen sulfide and nitric oxide are mutually dependent in the regulation of angiogenesis and endothelium-dependent vasorelaxation. Proc. Natl. Acad. Sci. U.S.A. 109, 9161–9166. 10.1073/pnas.1202916109 22570497 PMC3384190

[B11] Devkota S. Wang Y. Musch M. W. Leone V. Fehlner-Peach H. Nadimpalli A.. (2012). Dietary-fat-induced taurocholic acid promotes pathobiont expansion and colitis in Il10-/- mice. Nature 487, 104–108. 10.1038/nature11225 22722865 PMC3393783

[B12] Fan K. Li N. Qi J. Yin P. Zhao C. Wang L.. (2014). Wnt/beta-catenin signaling induces the transcription of cystathionine-gamma-lyase, a stimulator of tumor in colon cancer. Cell. Signal. 26, 2801–2808. 10.1016/j.cellsig.2014.08.023 25193114

[B13] Farrugia G. Szurszewski J. H. (2014). Carbon monoxide, hydrogen sulfide, and nitric oxide as signaling molecules in the gastrointestinal tract. Gastroenterology 147, 303–313. 10.1053/j.gastro.2014.04.041 24798417 PMC4106980

[B14] Fite A. Macfarlane G. T. Cummings J. H. Hopkins M. J. Kong S. C. Furrie E.. (2004). Identification and quantitation of mucosal and faecal desulfovibrios using real time polymerase chain reaction. Gut 53, 523–529. 10.1136/gut.2003.031245 15016746 PMC1774019

[B15] Gallego D. Clave P. Donovan J. Rahmati R. Grundy D. Jimenez M.. (2008). The gaseous mediator, hydrogen sulphide, inhibits *in vitro* motor patterns in the human, rat and mouse colon and jejunum. Neurogastroenterol. Motil. 20, 1306–1316. 10.1111/j.1365-2982.2008.01201.x 19019033

[B16] Goubern M. Andriamihaja M. Nubel T. Blachier F. Bouillaud F. (2007). Sulfide, the first inorganic substrate for human cells. FASEB J. 21, 1699–1706. 10.1096/fj.06-7407com 17314140

[B17] Gratzke C. Streng T. Waldkirch E. Sigl K. Stief C. Andersson K. E.. (2009). Transient receptor potential A1 (TRPA1) activity in the human urethra–evidence for a functional role for TRPA1 in the outflow region. Eur. Urol. 55, 696–704. 10.1016/j.eururo.2008.04.042 18468780

[B18] Hansen E. E. Lozupone C. A. Rey F. E. Wu M. Guruge J. L. Narra A.. (2011). Pan-genome of the dominant human gut-associated archaeon, Methanobrevibacter smithii, studied in twins. Proc. Natl. Acad. Sci. U.S.A. 108(Suppl. 1), 4599–4606. 10.1073/pnas.1000071108 21317366 PMC3063581

[B19] Hellmich M. R. Coletta C. Chao C. Szabo C. (2015). The therapeutic potential of cystathionine beta-synthetase/hydrogen sulfide inhibition in cancer. Antioxid. Redox Signal. 22, 424–448. 10.1089/ars.2014.5933 24730679 PMC4307161

[B20] Hellmich M. R. Szabo C. (2015). Hydrogen Sulfide and Cancer. Handb. Exp. Pharmacol. 230, 233–241. 10.1007/978-3-319-18144-8_12 26162838 PMC4665975

[B21] Hirata I. Naito Y. Takagi T. Mizushima K. Suzuki T. Omatsu T.. (2011). Endogenous hydrogen sulfide is an anti-inflammatory molecule in dextran sodium sulfate-induced colitis in mice. Dig. Dis. Sci. 56, 1379–1386. 10.1007/s10620-010-1461-5 20981572

[B22] Huycke M. M. Gaskins H. R. (2004). Commensal bacteria, redox stress, and colorectal cancer: mechanisms and models. Exp. Biol. Med. (Maywood). 229, 586–597. 15229352 10.1177/153537020422900702

[B23] Kashfi K. (2014). Anti-cancer activity of new designer hydrogen sulfide-donating hybrids. Antioxid. Redox Signal. 20, 831–846. 10.1089/ars.2013.5308 23581880 PMC3910473

[B24] Kashfi K. Olson K. R. (2013). Biology and therapeutic potential of hydrogen sulfide and hydrogen sulfide-releasing chimeras. Biochem. Pharmacol. 85, 689–703. 10.1016/j.bcp.2012.10.019 23103569 PMC3566320

[B25] Kimura H. (2011). Hydrogen sulfide: its production, release and functions. Amino Acids 41, 113–121. 10.1007/s00726-010-0510-x 20191298

[B26] Kimura Y. Goto Y. Kimura H. (2010). Hydrogen sulfide increases glutathione production and suppresses oxidative stress in mitochondria. Antioxid. Redox Signal. 12, 1–13. 10.1089/ars.2008.2282 19852698

[B27] Kleessen B. Kroesen A. J. Buhr H. J. Blaut M. (2002). Mucosal and invading bacteria in patients with inflammatory bowel disease compared with controls. Scand. J. Gastroenterol. 37, 1034–1041. 10.1080/003655202320378220 12374228

[B28] Leschelle X. Goubern M. Andriamihaja M. Blottiere H. M. Couplan E. Gonzalez-Barroso M. D.. (2005). Adaptative metabolic response of human colonic epithelial cells to the adverse effects of the luminal compound sulfide. Biochim. Biophys. Acta 1725, 201–212. 10.1016/j.bbagen.2005.06.002 15996823

[B29] Levitt M. D. Springfield J. Furne J. Koenig T. Suarez F. L. (2002). Physiology of sulfide in the rat colon: use of bismuth to assess colonic sulfide production. J. Appl. Physiol. (1985) 92, 1655–1660. 10.1152/japplphysiol.00907.2001 11896034

[B30] Li L. Rossoni G. Sparatore A. Lee L. C. Del Soldato P. Moore P. K. (2007). Anti-inflammatory and gastrointestinal effects of a novel diclofenac derivative. Free Radic. Biol. Med. 42, 706–719. 10.1016/j.freeradbiomed.2006.12.011 17291994

[B31] Linden D. R. Sha L. Mazzone A. Stoltz G. J. Bernard C. E. Furne J. K.. (2008). Production of the gaseous signal molecule hydrogen sulfide in mouse tissues. J. Neurochem. 106, 1577–1585. 10.1111/j.1471-4159.2008.05502.x 18513201 PMC2836856

[B32] Liu H. Bai X. B. Shi S. Cao Y. X. (2009). Hydrogen sulfide protects from intestinal ischaemia-reperfusion injury in rats. J. Pharm. Pharmacol. 61, 207–212. 10.1211/jpp.61.02.0010 19178768

[B33] Loubinoux J. Bronowicki J. P. Pereira I. A. Mougenel J. L. Faou A. E. (2002). Sulfate-reducing bacteria in human feces and their association with inflammatory bowel diseases. FEMS Microbiol. Ecol. 40, 107–112. 10.1111/j.1574-6941.2002.tb00942.x 19709217

[B34] Ma K. Liu Y. Zhu Q. Liu C. H. Duan J. L. Tan B. K.. (2011). H2S donor, S-propargyl-cysteine, increases CSE in SGC-7901 and cancer-induced mice: evidence for a novel anti-cancer effect of endogenous H2S? PLoS ONE 6:e20525. 10.1371/journal.pone.0020525 21738579 PMC3124470

[B35] Matsunami M. Tarui T. Mitani K. Nagasawa K. Fukushima O. Okubo K.. (2009). Luminal hydrogen sulfide plays a pronociceptive role in mouse colon. Gut 58, 751–761. 10.1136/gut.2007.144543 18852258

[B36] Medani M. Collins D. Docherty N. G. Baird A. W. O'Connell P. R. Winter D. C. (2011). Emerging role of hydrogen sulfide in colonic physiology and pathophysiology. Inflamm. Bowel Dis. 17, 1620–1625. 10.1002/ibd.21528 21674719

[B37] Modis K. Asimakopoulou A. Coletta C. Papapetropoulos A. Szabo C. (2013a). Oxidative stress suppresses the cellular bioenergetic effect of the 3-mercaptopyruvate sulfurtransferase/hydrogen sulfide pathway. Biochem. Biophys. Res. Commun. 433, 401–407. 10.1016/j.bbrc.2013.02.131 23537657

[B38] Modis K. Coletta C. Asimakopoulou A. Szczesny B. Chao C. Papapetropoulos A.. (2014). Effect of S-adenosyl-L-methionine (SAM), an allosteric activator of cystathionine-beta-synthase (CBS) on colorectal cancer cell proliferation and bioenergetics *in vitro*. Nitric Oxide 41, 146–156. 10.1016/j.niox.2014.03.001 24667534 PMC4156891

[B39] Modis K. Coletta C. Erdelyi K. Papapetropoulos A. Szabo C. (2013b). Intramitochondrial hydrogen sulfide production by 3-mercaptopyruvate sulfurtransferase maintains mitochondrial electron flow and supports cellular bioenergetics. FASEB J. 27, 601–611. 10.1096/fj.12-216507 23104984

[B40] Modis K. Panopoulos P. Coletta C. Papapetropoulos A. Szabo C. (2013c). Hydrogen sulfide-mediated stimulation of mitochondrial electron transport involves inhibition of the mitochondrial phosphodiesterase 2A, elevation of cAMP and activation of protein kinase A. Biochem Pharmacol. 86, 1311–1319. 10.1016/j.bcp.2013.08.064 24012591

[B41] Murata T. Sato T. Kamoda T. Moriyama H. Kumazawa Y. Hanada N. (2014). Differential susceptibility to hydrogen sulfide-induced apoptosis between PHLDA1-overexpressing oral cancer cell lines and oral keratinocytes: role of PHLDA1 as an apoptosis suppressor. Exp. Cell Res. 320, 247–257. 10.1016/j.yexcr.2013.10.023 24270013

[B42] Mustafa A. K. Gadalla M. M. Sen N. Kim S. Mu W. Gazi S. K.. (2009). H2S signals through protein S-sulfhydration. Sci. Signal. 2, ra72. 10.1126/scisignal.2000464 19903941 PMC2998899

[B43] Nagai Y. Tsugane M. Oka J. Kimura H. (2004). Hydrogen sulfide induces calcium waves in astrocytes. FASEB J. 18, 557–559. 10.1096/fj.03-1052fje 14734631

[B44] Ning N. Zhu J. Du Y. Gao X. Liu C. Li J. (2014). Dysregulation of hydrogen sulphide metabolism impairs oviductal transport of embryos. Nat. Commun. 5, 4107. 10.1038/ncomms5107 24914509

[B45] Oh G. S. Pae H. O. Lee B. S. Kim B. N. Kim J. M. Kim H. R.. (2006). Hydrogen sulfide inhibits nitric oxide production and nuclear factor-kappaB via heme oxygenase-1 expression in RAW264.7 macrophages stimulated with lipopolysaccharide. Free Radic. Biol. Med. 41, 106–119. 10.1016/j.freeradbiomed.2006.03.021 16781459

[B46] Patacchini R. Santicioli P. Giuliani S. Maggi C. A. (2005). Pharmacological investigation of hydrogen sulfide (H2S) contractile activity in rat detrusor muscle. Eur. J. Pharmacol. 509, 171–177. 10.1016/j.ejphar.2005.01.005 15733553

[B47] Picton R. Eggo M. C. Langman M. J. Singh S. (2007). Impaired detoxication of hydrogen sulfide in ulcerative colitis? Dig. Dis. Sci. 52, 373–378. 10.1007/s10620-006-9529-y 17216575

[B48] Pitcher M. C. Beatty E. R. Cummings J. H. (2000). The contribution of sulphate reducing bacteria and 5-aminosalicylic acid to faecal sulphide in patients with ulcerative colitis. Gut 46, 64–72. 10.1136/gut.46.1.64 10601057 PMC1727787

[B49] Pupo E. Pla A. F. Avanzato D. Moccia F. Cruz J. E. Tanzi F.. (2011). Hydrogen sulfide promotes calcium signals and migration in tumor-derived endothelial cells. Free Radic. Biol. Med. 51, 1765–1773. 10.1016/j.freeradbiomed.2011.08.007 21875664

[B50] Ramasamy S. Singh S. Taniere P. Langman M. J. Eggo M. C. (2006). Sulfide-detoxifying enzymes in the human colon are decreased in cancer and upregulated in differentiation. Am. J. Physiol. Gastrointest. Liver Physiol. 291, G288–G296. 10.1152/ajpgi.00324.2005 16500920

[B51] Rey F. E. Gonzalez M. D. Cheng J. Wu M. Ahern P. P. Gordon J. I. (2013). Metabolic niche of a prominent sulfate-reducing human gut bacterium. Proc. Natl. Acad. Sci. U.S.A. 110, 13582–13587. 10.1073/pnas.1312524110 23898195 PMC3746858

[B52] Roediger W. E. Moore J. Babidge W. (1997). Colonic sulfide in pathogenesis and treatment of ulcerative colitis. Dig. Dis. Sci. 42, 1571–1579. 10.1023/A:1018851723920 9286219

[B53] Rose P. Moore P. K. Ming S. H. Nam O. C. Armstrong J. S. Whiteman M. (2005). Hydrogen sulfide protects colon cancer cells from chemopreventative agent beta-phenylethyl isothiocyanate induced apoptosis. World J. Gastroenterol. 11, 3990–3997. 10.3748/wjg.v11.i26.3990 15996021 PMC4502092

[B54] Roudsari L. C. West J. L. (2015). Studying the influence of angiogenesis in *in vitro* cancer model systems. Adv. Drug Deliv. Rev. 97, 250–259. 10.1016/j.addr.2015.11.004 26571106

[B55] Rowan F. E. Docherty N. G. Coffey J. C. O'Connell P. R. (2009). Sulphate-reducing bacteria and hydrogen sulphide in the aetiology of ulcerative colitis. Br. J. Surg. 96, 151–158. 10.1002/bjs.6454 19160346

[B56] Scanlan P. D. Shanahan F. Marchesi J. R. (2009). Culture-independent analysis of desulfovibrios in the human distal colon of healthy, colorectal cancer and polypectomized individuals. FEMS Microbiol. Ecol. 69, 213–221. 10.1111/j.1574-6941.2009.00709.x 19496818

[B57] Sen N. Paul B. D. Gadalla M. M. Mustafa A. K. Sen T. Xu R.. (2012). Hydrogen sulfide-linked sulfhydration of NF-kappaB mediates its antiapoptotic actions. Mol. Cell 45, 13–24. 10.1016/j.molcel.2011.10.021 22244329 PMC3261430

[B58] Shatalin K. Shatalina E. Mironov A. Nudler E. (2011). H2S: a universal defense against antibiotics in bacteria. Science 334, 986–990. 10.1126/science.1209855 22096201

[B59] Shibuya N. Mikami Y. Kimura Y. Nagahara N. Kimura H. (2009a). Vascular endothelium expresses 3-mercaptopyruvate sulfurtransferase and produces hydrogen sulfide. J. Biochem. 146, 623–626. 10.1093/jb/mvp111 19605461

[B60] Shibuya N. Tanaka M. Yoshida M. Ogasawara Y. Togawa T. Ishii K.. (2009b). 3-Mercaptopyruvate sulfurtransferase produces hydrogen sulfide and bound sulfane sulfur in the brain. Antioxid. Redox Signal. 11, 703–714. 10.1089/ars.2008.2253 18855522

[B61] Singh S. Padovani D. Leslie R. A. Chiku T. Banerjee R. (2009). Relative contributions of cystathionine beta-synthase and gamma-cystathionase to H2S biogenesis via alternative trans-sulfuration reactions. J. Biol. Chem. 284, 22457–22466. 10.1074/jbc.M109.010868 19531479 PMC2755967

[B62] Stewart J. A. Chadwick V. S. Murray A. (2006). Carriage, quantification, and predominance of methanogens and sulfate-reducing bacteria in faecal samples. Lett. Appl. Microbiol. 43, 58–63. 10.1111/j.1472-765X.2006.01906.x 16834722

[B63] Suarez F. Furne J. Springfield J. Levitt M. (1998). Production and elimination of sulfur-containing gases in the rat colon. Am. J. Physiol. 274, G727–G733. 9575855 10.1152/ajpgi.1998.274.4.G727

[B64] Szabo C. Coletta C. Chao C. Modis K. Szczesny B. Papapetropoulos A.. (2013). Tumor-derived hydrogen sulfide, produced by cystathionine-beta-synthase, stimulates bioenergetics, cell proliferation, and angiogenesis in colon cancer. Proc. Natl. Acad. Sci. U.S.A. 110, 12474–12479. 10.1073/pnas.1306241110 23836652 PMC3725060

[B65] Szabo C. Hellmich M. R. (2013). Endogenously produced hydrogen sulfide supports tumor cell growth and proliferation. Cell Cycle 12, 2915–2916. 10.4161/cc.26064 23974103 PMC3875657

[B66] Szabo C. Papapetropoulos A. (2011). Hydrogen sulphide and angiogenesis: mechanisms and applications. Br. J. Pharmacol. 164, 853–865. 10.1111/j.1476-5381.2010.01191.x 21198548 PMC3195910

[B67] Szabo C. Ransy C. Modis K. Andriamihaja M. Murghes B. Coletta C.. (2014). Regulation of mitochondrial bioenergetic function by hydrogen sulfide. Part I. Biochemical and physiological mechanisms. Br. J. Pharmacol. 171, 2099–2122. 10.1111/bph.12369 23991830 PMC3976625

[B68] Tao B. B. Liu S. Y. Zhang C. C. Fu W. Cai W. J. Wang Y.. (2013). VEGFR2 functions as an H2S-targeting receptor protein kinase with its novel Cys1045-Cys1024 disulfide bond serving as a specific molecular switch for hydrogen sulfide actions in vascular endothelial cells. Antioxid. Redox Signal. 19, 448–464. 10.1089/ars.2012.4565 23199280 PMC3704125

[B69] Wallace J. L. Caliendo G. Santagada V. Cirino G. Fiorucci S. (2007a). Gastrointestinal safety and anti-inflammatory effects of a hydrogen sulfide-releasing diclofenac derivative in the rat. Gastroenterology 132, 261–271. 10.1053/j.gastro.2006.11.042 17241876

[B70] Wallace J. L. Dicay M. McKnight W. Martin G. R. (2007b). Hydrogen sulfide enhances ulcer healing in rats. FASEB J. 21, 4070–4076. 10.1096/fj.07-8669com 17634391

[B71] Wallace J. L. Vong L. McKnight W. Dicay M. Martin G. R. (2009). Endogenous and exogenous hydrogen sulfide promotes resolution of colitis in rats. Gastroenterology 137, 569–578.e1. 10.1053/j.gastro.2009.04.012 19375422

[B72] Wang J. Qiu B. Han L. Feng G. Hu Y. Chang L.. (2012). Effect of precursor and preparation method on manganese based activated carbon sorbents for removing H2S from hot coal gas. J. Hazard. Mater. 213–214, 184–192. 10.1016/j.jhazmat.2012.01.080 22341981

[B73] Wang R. (2009). Hydrogen sulfide: a new EDR*F*. Kidney Int. 76, 700–704. 10.1038/ki.2009.221 19536086

[B74] Wang R. (2012). Physiological implications of hydrogen sulfide: a whiff exploration that blossomed. Physiol. Rev. 92, 791–896. 10.1152/physrev.00017.2011 22535897

[B75] Whiteman M. Le Trionnaire S. Chopra M. Fox B. Whatmore J. (2011). Emerging role of hydrogen sulfide in health and disease: critical appraisal of biomarkers and pharmacological tools. Clin. Sci. 121, 459–488. 10.1042/CS20110267 21843150

[B76] Whiteman M. Li L. Rose P. Tan C. H. Parkinson D. B. Moore P. K. (2010). The effect of hydrogen sulfide donors on lipopolysaccharide-induced formation of inflammatory mediators in macrophages. Antioxid. Redox Signal. 12, 1147–1154. 10.1089/ars.2009.2899 19769459 PMC2875982

[B77] Wu D. Si W. Wang M. Lv S. Ji A. Li Y. (2015). Hydrogen sulfide in cancer: friend or foe? Nitric Oxide 50, 38–45. 10.1016/j.niox.2015.08.004 26297862

[B78] Wu Y. C. Wang X. J. Yu L. Chan F. K. Cheng A. S. Yu J.. (2012). Hydrogen sulfide lowers proliferation and induces protective autophagy in colon epithelial cells. PLoS ONE 7:e37572. 10.1371/journal.pone.0037572 22679478 PMC3362591

[B79] Yamagishi K. Onuma K. Chiba Y. Yagi S. Aoki S. Sato T.. (2012). Generation of gaseous sulfur-containing compounds in tumour tissue and suppression of gas diffusion as an antitumour treatment. Gut 61, 554–561. 10.1136/gutjnl-2011-300721 21836027

[B80] Yang G. Wu L. Jiang B. Yang W. Qi J. Cao K.. (2008). H2S as a physiologic vasorelaxant: hypertension in mice with deletion of cystathionine gamma-lyase. Science 322, 587–590. 10.1126/science.1162667 18948540 PMC2749494

